# Combined substituent number utilized machine learning for the development of antimicrobial agent

**DOI:** 10.1038/s41598-024-53888-2

**Published:** 2024-02-19

**Authors:** Keitaro Yamauchi, Hirotaka Nakatsuji, Takaaki Kamishima, Yoshitaka Koseki, Masaki Kubo, Hitoshi Kasai

**Affiliations:** 1 Institute of Multidisciplinary Research for Advance Materials (IMRAM), Tohoku University, Aoba-Ku, Sendai, Miyagi 980-8577 Japan; 2 East Tokyo Laboratory, Genesis Research Institute, Inc., 717-86 Futamata, Ichikawa, Chiba 272-0001 Japan; 3 Department of Chemical Engineering, Graduate School of Engineering, Tohoku University, Aoba-Ku, Sendai, Miyagi 980-8579 Japan

**Keywords:** Chemical biology, Computational biology and bioinformatics, Drug discovery, Molecular medicine, Chemistry, Mathematics and computing

## Abstract

The utilization of machine learning has a potential to improve the environment of the development of antimicrobial agents. For practical use of machine learning, it is important that the conversion of molecules information to an appropriate descriptor because too informative descriptor requires enormous computation time and experiments for gathering data, whereas a less informative descriptor has problems in validity. In this study, we utilized a descriptor only focused on substituent. The type and the position of substituents on the molecules that have a 4-quinolone structure (11,879 compounds) were converted to the combined substituent number (CSN). While the CSN does not include information on the detailed structure, physical properties, and quantum chemistry of molecules, the prediction model constructed by machine learning of CSN indicated a sufficient coefficient of determination (0.719 for the training dataset and 0.519 for the validation dataset). In addition, this CSN can easily construct the unknown molecules library which has a relatively consistent structure by recombination of substituents (32,079,318 compounds) and screening of them. The validity of the prediction model was also confirmed by growth inhibition experiments for *E. coli* using the model-suggested molecules and commercially available antimicrobial agents.

## Introduction

The development of new antimicrobial agents was continuously required due to the risk of the development of drug-resistant bacteria^1,2^. It is predicted that the number of resistant infections caused death will reach 10 million per year by 2050 without the development of novel antimicrobial agents^3^. However, the development environment of antimicrobial agents is getting worse and worse because of the depletion of potential drug targets, especially those from natural products^4^, the increase of the development cost associated with it, and lower profitability of them.

The utilization of informatics for the development of drugs and materials were not new^5,6^, but it has attracted more attention recently because of the development of computing capacity and computing algorithm including machine learning^7,8^. They enable the screening of candidate molecules of drugs by constructing a bioactivity prediction model from the accumulated training dataset. They have a potential to improve and accelerate the development environment of antimicrobial agents dramatically by decreasing the cost and saving time of drug discovery.

Machine learning constructs the prediction model by analyzing the correlation of data between target property and molecular information such as a molecular structure, physical property, and their calculated parameter of them. Thus, the information of the molecules needs to convert the descriptor which is mathematically analyzable data according to a specific rule to compute them^7,9,10^. One of the popular types of descriptors is key structure-based fingerprints. The key structure-based fingerprint represents the molecular structure by the presence of the key structure in pre-prepared list^11–13^. This type of fingerprint can effectively extract property-related information with a uniform data size. However, the validity of the calculation depends on the list of key structures because they don’t contain all the structural information of molecules. Therefore, they are sometimes utilized with topological path-based descriptors^14^ which can perceive all structure of the molecules to achieve high validity computation of molecules^15–17^. The highly informative and describable descriptor enables the construction of an effective prediction model and suggestion of the variety of candidate molecules. However, it also causes an increase in computation time and experiments to get the required information. In addition, it is also difficult to generate unknown candidate molecules because there is no limitation in size and structure. It often suggests an inconsistent structure. Therefore, it is still required an appropriate descriptor that can construct a prediction model that has enough accuracy without the requirement of a too-long computation time and too many experiments.

**Figure 1:**
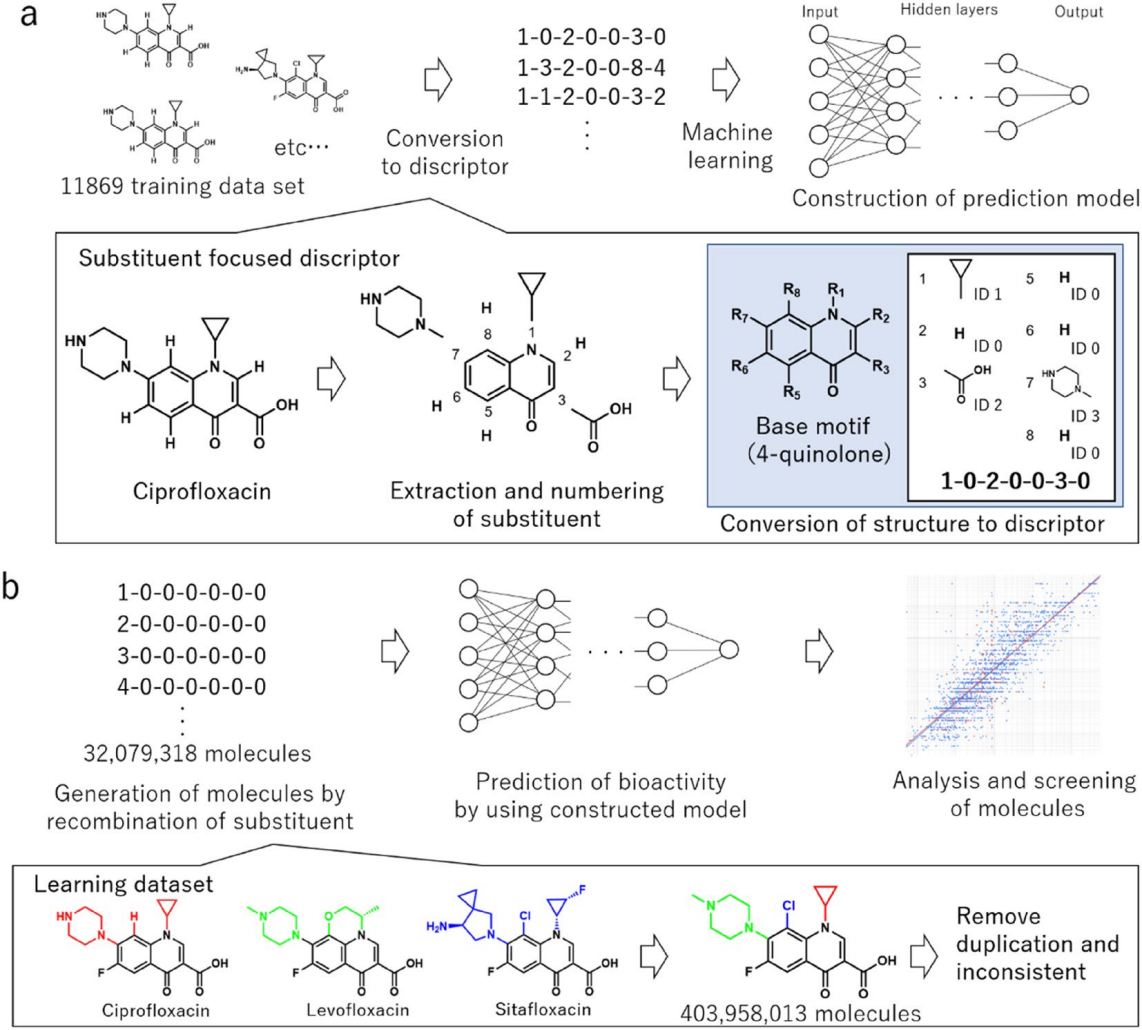
Schematic illustration of substituent focused machine learning and development of antimicrobial agent (**a**) Conversion of molecules which have 4-quinolone scaffold to substituent focused descriptor and construction of multi-layer perceptron model for prediction of antimicrobial activity by machine learning. (**b**) Analysis and screening of antimicrobial activity of the molecules generated by the recombination of substituent.

In this study, we focused on the type and the position of substituents on the scaffold of antimicrobial agents as the information of descriptor. In the field of drug development, it is an effective and popular method that investigates the bioactivity of molecules which has the same scaffold as the previously reported drugs but have a different substituent^18,19^. Therefore, it is considered that the substituent-focused descriptor is sufficient to construct the bioactivity prediction model by machine learning. The quinolone type antimicrobial agents were one of the most widely used antimicrobial agents. They have a 4-quinolone scaffold commonly and show antimicrobial activity against various bacteria without strong side effects^18^. Therefore, since the development of nalidixic acid in 1962, which is the first quinolone-type antimicrobial compound, quinolone-type antimicrobial agents were actively evaluated over 10,000 molecules, and over 40 molecules were launched in the market^18^. Thus, we focused on quinolone-type molecules as a target of machine learning to evaluate the validity of this descriptor. The information of the molecules with a 4-quinolone scaffold was converted to a combined substituent number (CSN) which is a descriptor only containing the information about the type and position of a substituent. The CSN utilized machine learning to construct prediction model of antimicrobial activity which have sufficient accuracy for training and validation dataset (Fig. 1a).

Moreover, the unknown molecule library was generated by the recombination of CSN (Fig. 1b). The recombination of CSN can generate a sufficient number of unknown molecules to screen the novel antimicrobial agents (32,079,318 compounds). In addition, the generated molecules have a consistent and relatively synthesizable structure because it is based on the previously reported substituents. The accuracy of the constructed model and the generated molecule library were confirmed by the growth inhibition experiments for the screened molecules. This descriptor is applicable to the development of other drugs and materials which have a common structure.

## Result and discussion

Initially, CheMBL^20^ database, which is the open database of bioactive molecules managed by European Bioinformatics Institute, was used to obtain the training dataset for machine learning (Fig. 1a). The molecules which have a 4-quinolone scaffold were screened from CheMBL and obtained minimal inhibition concentration (MIC) against *E. coli*. As a result, 11,869 molecules that have a 4-quinolone scaffold were extracted from CheMBL. Next, the 4-quinolone scaffold was removed from each structure information of molecule and the remained fragments were recorded separately for each substitution position. The recorded fragments numbered substituent ID in the order of appearance frequency in each substitution position (Fig. 1a below). In addition, the substituents that bind to multiple substitution positions were recorded with group information separately. At that time, the hydrated water and counter ion also counted as a free position fragment. Finally, the number of substituent types counted for each position were 155, 28, 113, 26, 25, 953, 59, and 14. From these processes, the molecular structure information was converted to substituent IDs arranged in the order of substituent position. The sequence of number was utilized as CSN. The original structure of each molecule can be reconstructed from this CSN and recorded substituent list. After that, the substituent ID was converted to a dummy variable and finally converted to 1373 bits of data. The extracted MIC were unit-aligned and converted to ordinary logarithms and stored with the combination of substituent ID. After that, we eliminated the compounds which have duplicated or have no data of MIC for *E. coli*. In this time, the recorded MIC that showed outliers, possibly due to recording errors, were also removed. From these processes, around 10,000 data were acquired for machine learning.

**Figure 2:**
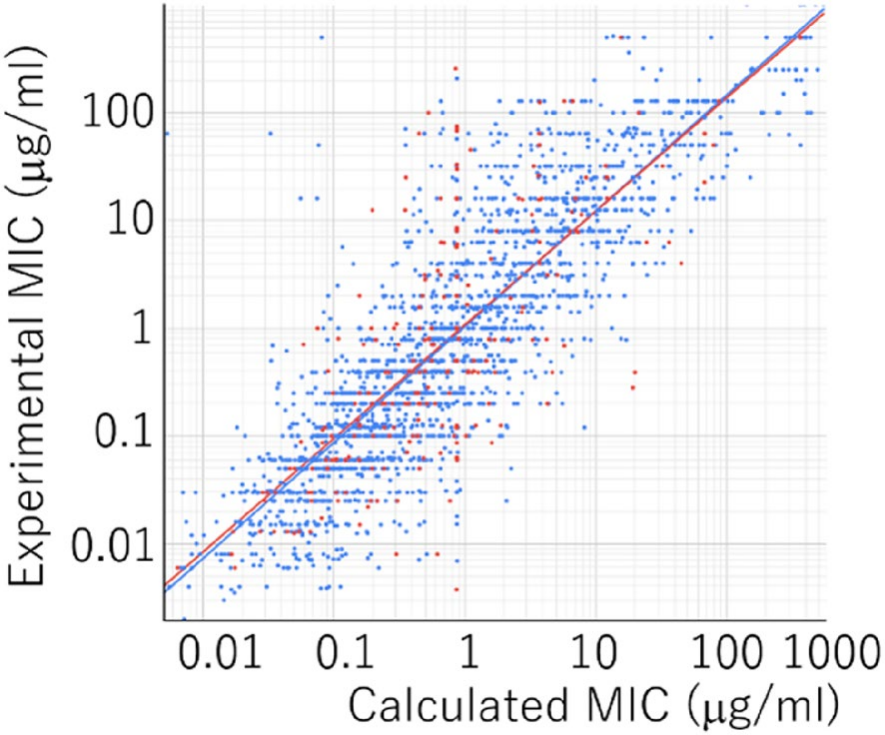
Plot of experimental versus predicted MIC for *E. coli* with the constructed prediction model. Blue dots indicated training dataset (*R*^2^ = 0.732). Red dots indicated validation dataset (*R*^2^ = 0.519).

The extracted data were randomly divided 9 to 1 into the training and validation dataset by using python script command in pandas library. Multi-Layer perceptron (MLP) was utilized as the learning algorithm^6,21^ for constructing the prediction model of MIC by using Keras models sequential, an open-source network library. MLP is one of the most popular models composed of several hidden layers, an input layer, and an output layer for machine learning. Each layer has several nodes and nonlinear functions connected them. In this research, the MLP model was constructed with multiple fully connected hidden layers with an input layer with the same dimensions as the CSN and single output. The hyperbolic tangent activation functions were utilized as the function to connect nodes. The elastic net regularization and dropout methods were added to avoid overfitting. The optimization of the model was performed by the root mean square propagation (RMSprop) algorithm. In this process, the number of hidden layers, the number of nodes in each layer, the learning rate of the RMSprop optimization algorithm, the parameters of elastic net regularization, the percentage of dropout, and the number of epochs to train the model were optimized to improve the validity of the model^22^ by OPTUNA™, which is an automatic hyperparameter optimization software framework. After optimization of the model, the coefficient of determination (*R*^2^) of the model calculated MIC and training data was increased at 0.732. This model also indicated a larger coefficient of determination against validation data (*R*^2^ = 0.519) than the general threshold of 0.5 (Fig. 2)^23^. Besides, the root mean square error (RMSE) for log_10_MIC was 0.385 for training and 0.501 for validation datasets. These data indicated this descriptor can construct a sufficient prediction model without demanding much information and long computation time.

**Figure 3:**
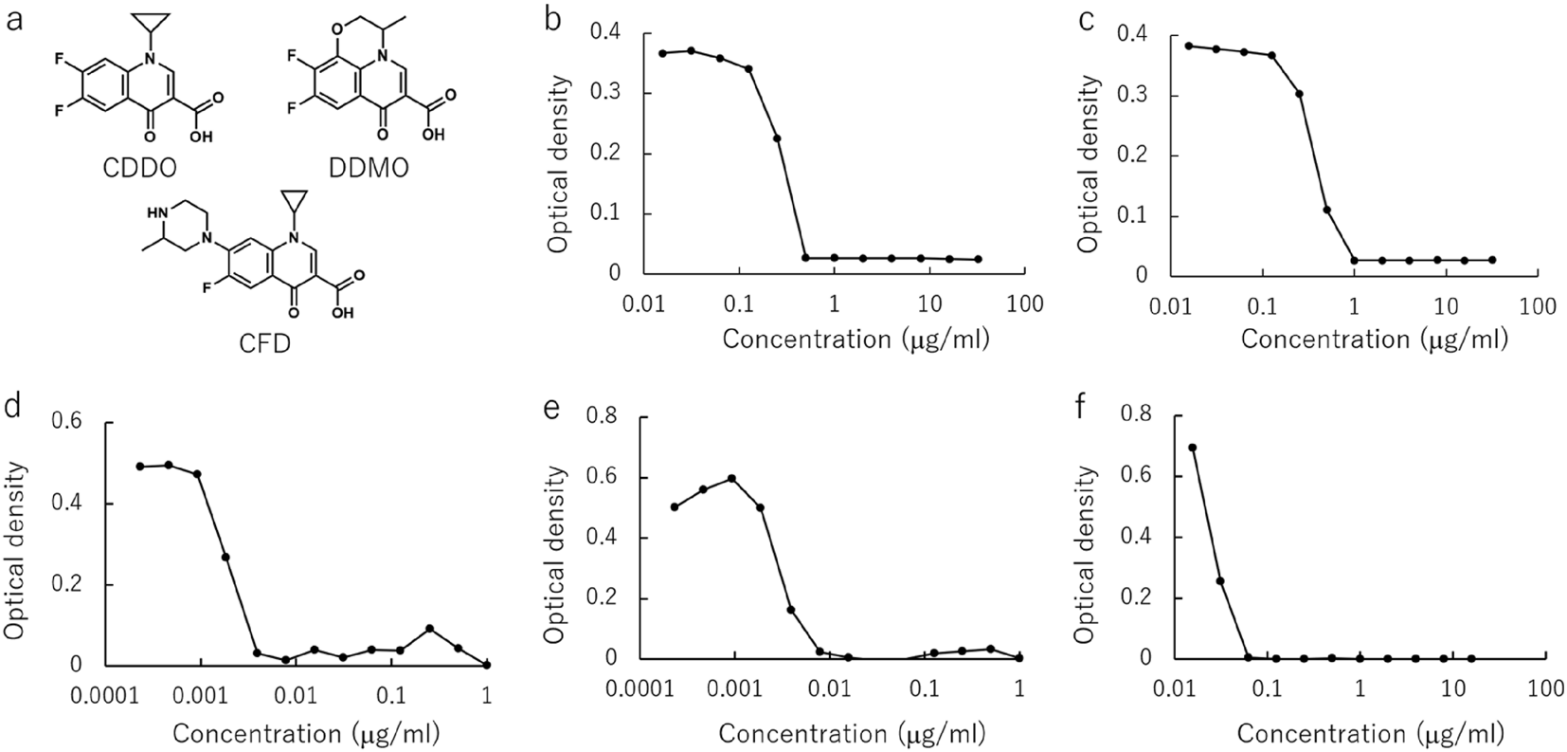
Structure (**a**) and concentration dependent growth inhibition activity for *E. coli* of (**b**) CDDO: 1-Cyclopropyl-6,7-difluoro-1,4-dihydro-4-oxoquinoline-3-carboxyalic acid (**c**) DDMO: 9,10-Difluoro-2,3-dihydro-3-methyl-7-oxo-7H-pyrido[1,2,3-*de*]-1,4-benzoxazine-6-carboxylic acid (**d**) CFD: 1-Cyclopropyl-6-fluoro-1,4-dihydro-7-(3-methyl-1-piperazinyl)-4-oxo-3-quinolinecarboxylic Acid (**e**) CPFX: ciprofloxacin (**f**) LVFX: levofloxacin.

Next, an unknown molecule library was generated by changing the combination of a substituent. All of the molecules were generated by recombining of substituent counted 4 or more times in the training dataset to improve reliability and limit excessive increase in the combinations. The recombination generated 32,079,318 molecules after excluding duplication and contradictory structure. The constructed model calculated the antimicrobial activity of generated molecule library. The growth inhibition experiments for *E. coli* was performed to confirm the validity of the prediction. The molecules which have CAS registry numbers but no MIC data for *E. coli* were screened from the calculated library and 50 molecules were founded in them. The growth inhibition activity of the 3 commercially available molecules in them (1-Cyclopropyl-6,7-difluoro-1,4-dihydro-4-oxoquinoline-3-carboxyalic acid : CDDO, 9,10-Difluoro-2,3-dihydro-3-methyl-7-oxo-7H-pyrido[1,2,3-*de*]-1,4-benzoxazine-6-carboxylic acid: DDMO, 1-Cyclopropyl-6-fluoro-1,4-dihydro-7-(3-methyl-1-piperazinyl)-4-oxo-3-quinolinecarboxylic Acid: CFD) were compared with Levofloxacin (LVFX)^24^ and Ciprofloxacin (CPFX)^25^ (Fig. 3 and S1, Table 1). As shown in the Table 1, experimentally measured MIC showed a similar tendency to predicted MIC. It indicated our constructed model and calculated data library are efficient for the developing antimicrobial agents.

**Table 1:** Experimental MIC and predicted MIC of antimicrobial agents and screened molecules.

	Experimental	Predicted
CDDO	0.50	0.634
DDMO	1.0	0.848
CFD	0.007	0.084
CPFX	0.008	0.114
LVFX	0.064	0.444

*CDDO* 1-Cyclopropyl-6,7-difluoro-1,4-dihydro-4-oxoquinoline-3-carboxyalic acid, *DDMO* 9,10-Difluoro-2,3-dihydro-3-methyl-7-oxo-7H-pyrido[1,2,3-*de*]-1,4-benzoxazine-6-carboxylic acid, *CFD* 1-Cyclopropyl-6-fluoro-1,4-dihydro-7-(3-methyl-1-piperazinyl)-4-oxo-3-quinolinecarboxylic Acid, *CPFX* ciprofloxacin, *LVFX* levofloxacin.

The structures of molecules calculated to have the highest antimicrobial activity in the library are shown in Figure S2. It was found that the molecules commonly have the same combination of substituents in each position. However, further screening of the molecules was conducted because it is difficult to synthesize the molecules which have 2-amino-3, 5-fluoropyridine at the 2 position, and 3-amino-4-ethylpiperidine at the 7 position. To help the screening of the substituent, the influence of the substituent can be estimated by analyzing the calculated library. The distribution of predicted antimicrobial activity of molecules that have trifluoromethyl group or proton at 2-position were indicated in Figure S3. These data indicated trifluoromethyl group has a strong influence on antimicrobial activity. Thus, the calculated library is useful to find suitable antimicrobial agent candidates by estimating the influence of each substituent and its combination on the calculated antimicrobial activity and the difficulty of preparation.

This CSN can be classified into a key structure-based fingerprint. In this method, CSN creates a key structure list dedicated for the target molecular group. Therefore, the CSN is expected to maintain the same performance even for other molecular groups because of a specific key structure list for each molecular group. Certainly, the CSN is difficult to contribute to developing the major structure change such as scaffold hopping^26^ and the discovery of the new working mechanisms of drugs because the main scaffold of learning data set was limited and the detailed structure of molecules were not calculated. However, CSN can generate efficient number of molecules which is relatively easy to synthesize because they are based on previously reported substituents. In addition, the low requirement of data and computation cost enabled the easy utilization of previously accumulated data. Therefore, CSN can especially contribute to the matured research field.

In summary, this research proposed a descriptor focused on the type and substitution position on the main scaffold of an antimicrobial agent. The prediction model of antimicrobial activity was constructed by machine learning of the molecules information which commonly has a 4-quinolone structure collected from CheMBL. Even though the descriptor does not include detailed information such as physical properties and quantum chemistry of molecules, the prediction model indicated a high coefficient of determination (0.719 for the learning data set and 0.519 for the validation data set). The growth inhibition experiments for *E. coli* also confirmed the validity of the model. In addition, the new candidate molecules of antimicrobial agents which have relatively rational structures were generated through the recombination of substituent combinations. The descriptor in this research enables the easy introduction of machine learning into the development of drugs and materials which have a common scaffold, due to the less information requirement and construction of sufficient prediction model without high computational cost. Currently, society is working towards the use of informatics in various fields. However, the requirement of many experiments and the incompleteness of the accumulated information has prevented it. We believe that this descriptor could contribute to remove the barriers to the introduction of informatics and strongly support matured research field.

## Materials and methods

### Materials

1-Cyclopropyl-6,7-difluoro-1,4-dihydro-4-oxoquinoline-3-carboxyalic acid and 9,10-Difluoro-2,3-dihydro-3-methyl-7-oxo-7H-pyrido[1,2,3-*de*]-1,4-benzoxazine-6-carboxylic acid were purchased from Tokyo chemical Industry (Tokyo, Japan). Levofloxacin, ciprofloxacin and 1-Cyclopropyl-6-fluoro-1,4-dihydro-7-(3-methyl-1-piperazinyl)-4-oxo-3-quinolinecarboxylic Acid were purchased from Nacalai Tesque (Kyoto, Japan). The utilized computer was composed of CPU: AMD Ryzen Threadripper 3960X, GPU; NVDIA GeForce RTX 2080 Ti×2, RAM 64 GB, SSD; 500 GB and OS; Debian 10 buster. *E. coli* (ATCC25922) was purchased from Microbiologics (MS, USA). Standard Methods Agar “DAIGO” was purchased from Fujifilm Wako (Tokyo, Japan). ChEMBL26 (https://www.ebi.ac.uk/chembl/) was utilized as the database of bioactive molecules. The extracted data was managed by SQLite (https://www.sqlite.org/index.html), RDKit (https://rdkit.org/docs/index.html), Numpy (https://numpy.org) and Pandas (https://pandas.pydata.org/). Machine learning were performed by using keras sequential model (https://keras.io/ja/). Optimization of hyperparameter of model were performed by Optuna (https://optuna.org/).

### Methods

#### Model training and prediction

The training dataset was obtained from CheMBL^20^. The chemical structures of the molecules were acquired as SMILES using SQLite script. The extraction of the compounds that have a 4-quinolone scaffold was performed by using RDKit. At first, the MDL mol file of the 4-quinolone scaffold was prepared. The acquired SMILES data were read, and extracted the compounds which have 4-quinolone structure by substructure searching of them by using the MDL mol file of 4-quinolone. The 4-quinolone scaffold was removed from the extracted molecule structure to make the fragments of substituents on the 4-quinolone scaffold. The structures of the remaining fragments were recorded separately for each substitution position. At first, if the fragment was different from the recorded structure, the structure of the fragment was recorded as a new substituent, and the number of substituent counts was set to 1. And, if it matched the recorded structure, the number of the substituent count of it was increased by one. After recording all the fragments of extracted compounds, they were sorted in the number of the substructure count. The order of the substituent count number was used as the substituent ID. In this time, the substituents connected to multiple scaffold positions were also recorded as the grouped information. The hydrated water and counter ion were counted as a free position substituent. Finally, each substituent in the compound was converted to a numerical sequence of substituent IDs in the order of substituent position of the 4-quinolone.

The MIC of the obtained molecule data was collected with unifying the unit as µg/ml. The molecules were dropped from the data set as an outlier if the MIC were higher than 10,000 µg/ml or lower than 0.001 µg/ml. If the multiple MIC data were recorded in CheMBL for one molecule, the median value was adopted. The extracted MIC were converted to ordinary logarithms and stored with the combination of substituent ID. The substituent ID was converted to a dummy variable. The data were randomly divided 9 to 1 into the training and validation dataset by using pandas library. In this time, the seed of random division is fixed to 123. The dense MLP model for MIC prediction was constructed by Keras model sequential. The number of nodes in the first layer was set to the number of dimensions as the CSN. After that, the hidden layers were added where the number of layers and nodes are variable. At this time, the Keras library utilized elastic net regularization and dropout methods were used for the model to avoid overfitting. The model optimization was performed by “keras.optimizer.RMSprop”. Optimization of its hyperparameter (the number of hidden layers, the number of nodes, the learning rate of RMSprop optimization algorithm, the parameters of elastic net regularization, the percentage of dropout, number of epochs to train the model) was performed by using OPTUNA™. *R*^2^ was evaluated for the training dataset and the validation dataset each other. The substituents with a count of 3 or less were excluded from the list of substituents. After that, all combination of the substituents were generated by “meshgrid” in NumPy. After that, inconsistent combinations of fragments bound to multiple substitution position were eliminated from the combinations. The optimized prediction model calculated the generated combination.

#### Growth inhibition assay

Freeze stock of *E. coli* (ATCC25922) were seeded on Standard Agar medium and incubated at 37 °C over night. *E. coli* colonies are picked and cultured in LB medium at 37 °C for several hours. The cultured *E. coli* dispersion was diluted by PBS at OD_600_ 0.2. 25 μl of the solution was mixed with 12 ml of LB medium and added in a 96 well plate at 50 μl/well. 50 μl of the antimicrobial agent solution in each concentration was added to a 96 well plate and incubated at 37 °C for 24 h. OD_600_ of a 96 well plate was measured by plate reader (iMARK, Bio-rad, CA, USA).

### Supplementary Information

Supplementary Information.

## Data Availability

The datasets used and analyzed during the current study are available from the corresponding author on reasonable request.
