# Supplementary material for: Combined substituent number utilized machine learning for the development of antimicrobial agent
Source: Sci Rep. 2024 Feb 19;14:4106. doi: 10.1038/s41598-024-53888-2 (PMC10876936; doi:10.1038/s41598-024-53888-2)
Supplement: Supplementary file 1 — Supplementary Information. [file 41598_2024_53888_MOESM1_ESM.docx]

Supplementary Information

**Combined Substituent Number Utilized Machine Learning for the Development of Antimicrobial Agent**

**Keitaro Yamauchi^1^, Hirotaka Nakatsuji^1,2^*, Takaaki Kamishima^2^, Yoshitaka Koseki^1^, Masaki Kubo^3^, Hitoshi Kasai^1^***

^1^Institute of Multidisciplinary Research for Advance Materials (IMRAM) Tohoku University, Aoba-ku, Sendai, Miyagi, 980-8577, Japan

^2^Genesis research institute, Inc. East Tokyo laboratory, 717-86 Futamata, Ichikawa, Chiba 272-0001, Japan

^3^Department of Chemical Engineering, Graduate school of Engineering, Tohoku University, Aoba-ku, Sendai, Miyagi, 980-8579, Japan

Email:

Hirotaka Nakatsuji: hirotaka.nakatsuji.d1@tohoku.ac.jp

Hitoshi Kasai: kasai@tohoku.ac.jp


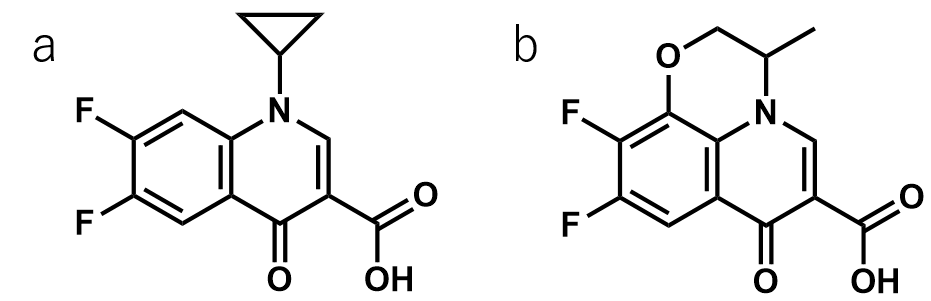


**Figure S1.** Structure of CPFX and LVFX.


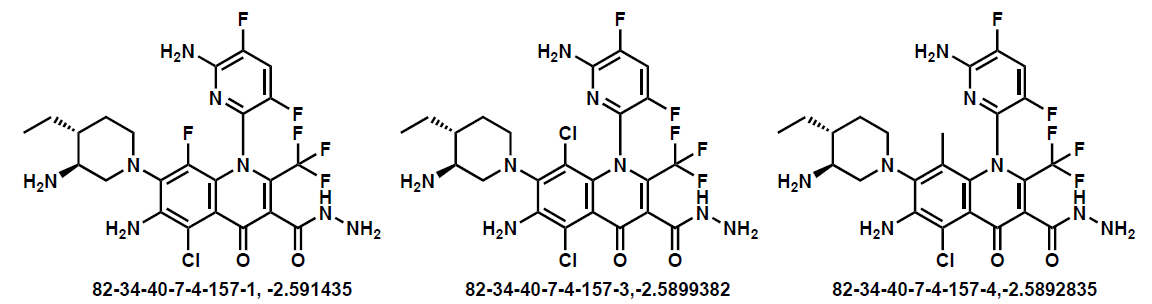


**Figure. S2** Structure of the molecules predicted to have the highest antimicrobial activity.


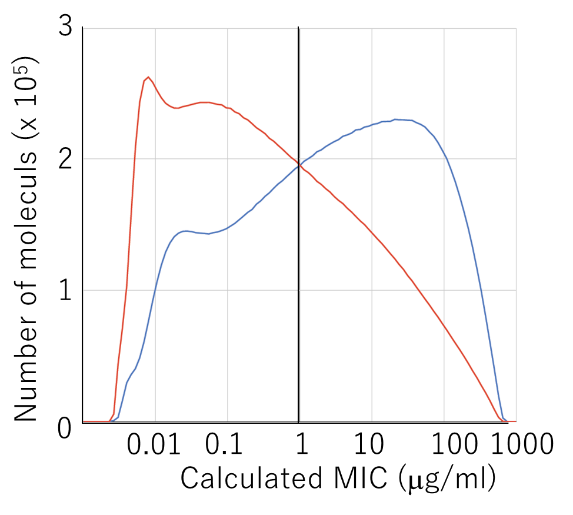


**Figure S3**. Prediction of the effect of trifluoromethyl group at 2- position for MIC against *E. coli*. Blue line indicated the calculated MIC of the molecules which have trifluoromethyl group at 2- position. Red line indicated that have no substituent at 2- position.
